# Bromodomain and Extra Terminal (BET) Inhibitor Suppresses Macrophage-Driven Steroid-Resistant Exacerbations of Airway Hyper-Responsiveness and Inflammation

**DOI:** 10.1371/journal.pone.0163392

**Published:** 2016-09-22

**Authors:** Thi Hiep Nguyen, Steven Maltby, Fiona Eyers, Paul S. Foster, Ming Yang

**Affiliations:** Priority Research Centre for Healthy Lungs, School of Biomedical Sciences & Pharmacy, Faculty of Health and Medicine and Hunter Medical Research Institute, The University of Newcastle, Callaghan, NSW 2300, Australia; University of Kentucky, UNITED STATES

## Abstract

**Background:**

Exacerbations of asthma are linked to significant decline in lung function and are often poorly controlled by corticosteroid treatment. Clinical investigations indicate that viral and bacterial infections play crucial roles in the onset of steroid-resistant inflammation and airways hyperresponsiveness (AHR) that are hallmark features of exacerbations. We have previously shown that interferon γ (IFNγ) and lipopolysaccharide (LPS) cooperatively activate pulmonary macrophages and induce steroid-resistant airway inflammation and AHR in mouse models. Furthermore, we have established a mouse model of respiratory syncytial virus (RSV)-induced exacerbation of asthma, which exhibits macrophage-dependent, steroid-resistant lung disease. Emerging evidence has demonstrated a key role for bromo- and extra-terminal (BET) proteins in the regulation of inflammatory gene expression in macrophages. We hypothesised that BET proteins may be involved in the regulation of AHR and airway inflammation in our steroid-resistant exacerbation models.

**Methodology/Principal Findings:**

We investigated the effects of a BET inhibitor (I-BET-762) on the development of steroid-resistant AHR and airway inflammation in two mouse models. I-BET-762 administration decreased macrophage and neutrophil infiltration into the airways, and suppressed key inflammatory cytokines in both models. I-BET treatment also suppressed key inflammatory cytokines linked to the development of steroid-resistant inflammation such as monocyte chemoattractant protein 1 (MCP-1), keratinocyte-derived protein chemokine (KC), IFNγ, and interleukin 27 (IL-27). Attenuation of inflammation was associated with suppression of AHR.

**Conclusions/Significance:**

Our results suggest that BET proteins play an important role in the regulation of steroid-resistant exacerbations of airway inflammation and AHR. BET proteins may be potential targets for the development of future therapies to treat steroid-resistant inflammatory components of asthma.

## Introduction

Exacerbations of asthma are defined as a worsening of clinical symptoms such as wheeze, shortness of breath, chest tightness, bronchial hyper-responsiveness and airflow obstruction and can occur in all asthmatic patients, regardless of disease severity [1, 2]. Exacerbations are a major cause of hospitalisation, impaired long-term lung function and account for significant healthcare costs related to asthma [3, 4]. Although mild to moderate asthma can generally be well-controlled with corticosteroid treatment, exacerbations are often difficult to manage with corticosteroids and new effective approaches to treatment are needed.

Respiratory viral infections are major triggers of asthma exacerbations and bacterial infections can also worsen disease [3, 5]. Allergic asthma is associated with activation of type 2 immune responses (CD4+ T-helper type 2 lymphocytes (Th2 cells) and type 2 innate lymphoid cells (ILC2) and increased airway eosinophil numbers. Viral-induced exacerbations may reinforce the type 2 response and/or drive activation of innate host defence responses in conjunction with exaggerated T helper cell-regulated responses (e.g. Th1, Th2 and Th17 cells) [5, 6]. In particular, neutrophils are prominent in cellular infiltrates and increased pro-inflammatory cytokines (e.g. IL-8, TNFα, IFNγ and MCP-1) are a feature of acute exacerbations in asthmatic patients following viral and bacterial infections [5–9]. The mechanisms that underpin viral-induced exacerbations and the subsequent development of steroid-resistance remain poorly understood.

Recent clinical studies suggest that innate immune cell activation in the lung (e.g. macrophages and neutrophils) underpins the pathogenesis of steroid-resistant asthma [5, 10, 11]. Further, emerging findings in animal models suggest that macrophages play a key role in the induction of steroid-resistant airway inflammation and AHR [12, 13]. Our laboratory has demonstrated that macrophage activation by IFNγ and LPS regulates steroid-resistant AHR and airway inflammation through IL-27 and myeloid-differentiation-primary-response-gene 88 (MyD88)-dependent pathways [12, 14]. Importantly, IFNγ or LPS alone do not induce these features of disease and cooperative signalling between IFNγ and LPS/TLR4/MyD88 pathways is required to induce steroid resistant inflammation and AHR [14]. The expression of IFNγ, IL-27 and TNFα are all increased in sputum from steroid-resistant neutrophilic asthma patients where viral and bacterial infections have been implicated in exacerbations and disease progression [12, 15]. Although increasing levels of IFNγ and LPS (endotoxin) are directly associated with severity of asthma [16, 17], and investigations with these factors demonstrate that they drive critical pathogenic mechanisms linked to exacerbations, they do not replicate the complexity of infection. Recently, we have extended our modelling and developed a model of viral-induced exacerbations of asthma. We have demonstrated that respiratory syncytial virus (RSV) infection exacerbates AHR and airway inflammation in mice with pre-existing allergic airways disease [15] mimicking human disease. RSV-induced exacerbations were characterized by increased macrophage and neutrophil infiltration into the lung, elevated levels of MCP-1 and TNFα in the lung, and steroid-resistant pathology including induction of AHR [15]. Schneider *et al*. have also reported that pulmonary macrophages can regulate the development of AHR by producing MCP-1 in acute rhinovirus-induced exacerbations of allergic airways disease [18]. Increased pulmonary macrophage numbers have also been reported in non-eosinophilic asthma, in two clinical studies [10, 19]. Therefore, findings in both human and animal investigations suggest that pulmonary macrophages may play an important role in steroid-resistant exacerbations of asthma.

Recently, a role for histone acetylation has been demonstrated in the regulation of inflammation, and this mechanism may also operate in various inflammatory diseases of the lung (e.g. asthma and chronic obstructive pulmonary disease) [20, 21]. Histone acetylation is recognised by bromo- and extra-terminal (BET) protein family members, including bromodomains (BRD)-2, BRD3, and BRD4 [22, 23]. Administration of BET inhibitors, including I-BET-762 (GSK-525762A) and JQ1, can dampen the upregulation of inflammatory gene expression [24–26]. I-BET-762 inhibits the binding of acetylated lysines by BET proteins, disrupting chromatin complexes essential for inflammatory gene expression [25]. I-BET-762 also regulates T cell differentiation by altering expression of c-myc, a transcriptional regulator expressed during T-cell activation, but appears to have no effect on the function of fully matured T cells [27]. Recently, I-BET-762 was also shown to inhibit asthmatic smooth muscle cell proliferation and production of inflammatory mediators (e.g. IL-6 and IL-8) [28]. IL-8 is the human homolog of mouse keratinocyte-derived protein chemokine (KC), and both molecules act as neutrophil chemoattractants. I-BET-762 treatment has also been shown to inhibit the production of multiple pro-inflammatory cytokines (e.g. IL-1β, IL-12α, IL-6 and IFN-β) in LPS-stimulated bone marrow-derived macrophages (BMDMs) [25]. Further, in mouse models of lethal sepsis and *Helicobacter pylori* infection BET inhibition dampens inflammation and protects from disease [25, 26].

Collectively, these observations demonstrating the proinflammatory role of BET proteins led us to hypothesise that these molecules may play important roles in the regulation of airway inflammation and AHR in steroid-resistant exacerbations of asthma. In our study, we assessed the impact of the BET inhibitor, I-BET-762, on the development of IFNγ/LPS and RSV-induced steroid-resistant airway inflammation and AHR. In both models, I-BET-762 treatment suppressed the development AHR, macrophage and neutrophil infiltration and the expression of MCP-1 and KC in the lung. I-BET-762 treatment also inhibited the expression of pro-inflammatory cytokines, including those linked to the development of steroid resistance (e.g. IL-27, IFNγ, and TNFα) in our model of RSV-induced exacerbation.

## Materials and Methods

### Ethics statement

This study was carried out in strict accordance with the requirements of the Australian Code of Practice for the Care and Use of Animals for Scientific Purposes. All protocols were approved by the Animal Care and Ethics Committee of The University of Newcastle, Australia (Permit numbers A-2011-165 and A-2013-337).

### Mice and animal welfare

Specific pathogen-free BALB/c male mice (6–8 weeks old) were obtained from The University of Newcastle Animal Services Unit. Mice were housed in plastic cages with adsorbent bedding and controlled temperature and light dark cycle (12h: 12h) under specific pathogen-free facilities in the Bioresources Facility, Hunter Medical Research Institute (Newcastle, Australia). Mice were provided with water and food *ad libitum* and acclimatised for one week prior to experimentation. All treated mice were monitored daily as part of the approved protocol. There were no animal deaths or interventions required as a result of our protocols. At the end of each experimental time point, animals were sacrificed by sodium pentobarbitone injection (Virbac, Australia), and all efforts were made to minimize suffering in treated mice.

### RSV production

As previously described [29], human RSV (long strain, type A) was propagated in monolayers of Hep-2 cells for 4 days. Supernatants and cell lysates were collected in 50mM HEPES and 100mM MgSO_4_. Virus was purified using two-step sucrose gradients (33% and 77%) by ultracentrifugation at 100,000g for 1.5h. RSV titre was quantified by plaque assay using Hep-2 cells.

### IFNγ/LPS-induced AHR and airway inflammation

Mice were anesthetized with 100μl Alfaxan solution diluted (1:4) with sterile PBS by intravenous (i.v.) injection. Murine recombinant IFNγ (1.5μg/mouse; PeproTech), LPS (50ng/mouse; InvivoGen), and mouse recombinant MCP-1 (rMCP-1) (50ng/mouse; PeproTech) were administered intratracheally (i.t.), as previously described [30]. Where indicated, mice were treated with I-BET-762 (30mg/kg; Merk Millipore) or vehicle (20% beta-cyclodextrin, 2% DMSO in 0.9% saline) i.v. 1h before and 4h after exposure to IFNγ/LPS and endpoints were assessed 12h after IFNγ/LPS administration.

### Pulmonary macrophage isolation

Pulmonary macrophages were isolated from naïve mouse lungs as previous described [15]. Briefly, cells were mechanically isolated from mouse lung tissues through 70um cell strainers. Macrophages then were purified by gradient centrifugation (Histopaque 1083; Sigma-Aldrich) and seeded at a concentration of 1×10^6^ cells/well in Dulbecco’s Modified Eagle Medium (DMEM) (Sigma-Aldrich) containing 20% fetal bovine serum (FCS). After 3h, all non-adherent cells were removed and >95% of adherent cells were macrophages. Isolated macrophages were then stimulated with PBS or LPS (50ng/ml; Sigma-Aldrich) and IFNγ (1.5μg/ml; Pepro Tech) in the presence of I-BET-762 (10μM; Merck Millipore) or vehicle (20% hydroxyl-β cyclodextrin, 2% DMSO in 0.9% saline) (Sigma, Aldrich) for 12h, then harvested into Trizol for mRNA qualification.

### RSV-induced exacerbations of underlying allergic airways disease

As previously described [15], mice were initially sensitised to ovalbumin (OVA) (50μg/mouse) or PBS in 1mg Alhydrogel (alum) on day 0 by intraperitoneal injection (i.p.). Mice were then challenged with 1% OVA aerosol in saline daily for 30 min/day from day 13 to 16. Where indicated, mice were exposed to RSV (0.5×10^6^ Plaque-Forming Units (PFU)/mouse) by intranasal (i.n.) administration on day 19. On day 23, following induction of allergic disease, some mice were treated with I-BET-762 (30mg/kg) or vehicle twice (every 12 hours) i.v. Airway inflammation and AHR were assessed on day 24 (5 days post-infection).

### Measurement of lung function (AHR)

Airway resistance (Raw) in response to increasing concentrations of methacholine (MCh, Sigma-Aldrich) was measured using a flexivent apparatus (FX1 system, Scireq; Montreal, Canada), as previously described [12, 31]. Briefly, mice were anesthetised with 150 microliters of a mixture containing xylazine (10 mg/kg) and ketamine (100 mg/kg; Troy Laboratories, Smithfield, Australia). Mice were ventilated with a tidal volume of 8 ml/kg at a rate of 450 breaths/min. Airway resistance is presented as percentage increase over baseline (saline).

### Broncheoalveolar lavage fluid (BALF)

BALF was collected and processed as previously described [15]. Briefly, the left lung was tied off and the right lung was washed twice with sterile Hank’s Balanced Salt Solution (HBSS). Cells was collected and resuspended in red blood cell lysis buffer. Total cell counts were determined by haemocytometer and remaining cells were cytocentrifuged onto glass slides (ThermoFisher Scientific, Scoresby, Victoria, Australia). BALF cells were stained with May-Grunwald-Giemsa, and differential leukocyte counts were performed based on morphological criteria by light microscopy.

### RNA isolation, reverse-transcription (RT)-PCR and quantitative PCR (qPCR)

RNA was isolated from cells or lung tissue in TRIZOL RNA isolation buffer according to manufacturer’s instructions (Invitrogen, Mount Waverly, Victoria, Australia). cDNA was prepared by RT-PCR using random hexamer primers (Invitrogen) on a T100 thermal cycler (Bio-Rad). Q-PCR was performed on a Viia7 real-time PCR machine (Life Technologies) using SYBR reagents. The mRNA levels were calculated using 2^−ΔΔC*t*^ relative to the reference gene hypoxanthine-guanine phosoribosyl-transferase (HPRT) (internal control) and expressed as a fold change relative to control samples. Primers used were HPRT- Fwd 5’-aggccagactttgttggatttgaa-3’, Rev 5’-caacttgcgctcatcttaggcttt-3’; TNFα Fwd 5’-tctgtctactgaacttcggggtga-3’, Rev 5’-ttgtctttgagatccatgccgtt-3’; MCP-1 Fwd 5’-ccaactctcactgaagccagctct-3’, Rev 5’-tcagcacagacctctctcttgagc-3’; KC Fwd 5’-caatgagctgcgctgtcagtg-3’, Rev 5’-cttggggacaccttttagcatc-3’; IL-27p28 Fwd 5’-gcagcctcctagcctttgtg-3’, Rev 5’-ggagtcggtacttgagagagaag-3’; IFNγ Fwd 5’-atgaacgctacacactgcatc-3’, Rev 5’-gctgatggcctgattgtcttt-3’. RSV levels were quantified using plasmid copy number standards for the RSV N gene and HPRT with the following RSV N gene primers Fwd 5’-catccagcaaatacaccatcca-3’, Rev 5’-ttctgcacatcataattaggagtatcaa-3’.

### Protein Quantification

Lung tissues were homogenized in radioimmunoprecipitation assay buffer (Sigma-Aldrich) containing a protease/phosphatase inhibitor cocktail (Cell Signalling Technology; Danvers, MA) on a Tissuelyser LT tissue disruptor (Qiagen; Valencia, CA). Recovered supernatants were used to measure protein levels. Protein concentrations (MCP-1, KC, IFNγ, IL-27p28 and TNFα) in lung homogenates were determined by ELISA (R&D systems), according to the manufacturer’s instruction. MCP-1 and TNFα levels in the RSV model were measured using a cytometric bead array (CBA) kit according to the manufacturer’s instruction (BD Biosciences) as previously described [15]. All lung protein data have been normalized to total protein levels (pictograms per milligram of total protein).

### Statistics

All data was analysed using GraphPad Prism 6 Software (San Diego, CA, USA) and presented as mean ± S.E.M. Airway resistance data were analysed by two-way ANOVA followed by a Bonferroni correction for comparisons between multiple experimental groups. Other data including different cell counts, mRNA and protein levels data were analysed using the unpaired Mann-Whitney test. Differences were considered statistically significant if P<0.05.

## Results

### I-BET-762 administration inhibits the development of steroid resistant inflammation and AHR in IFNγ/LPS-treated mice

**Fig 1:**
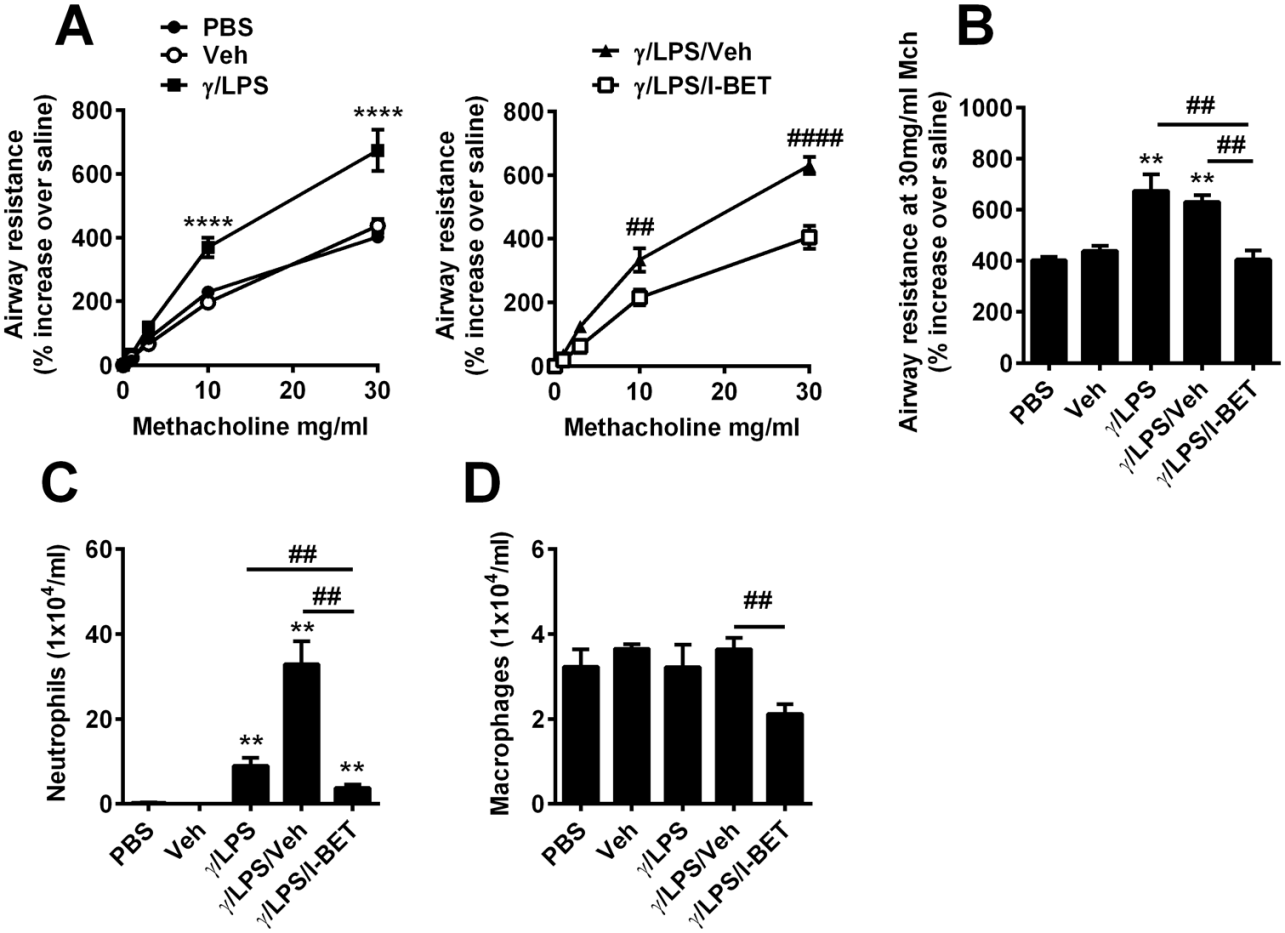
I-BET-762 treatment suppresses AHR and inflammatory cells after IFNγ/LPS stimulation. Mice were administered IFNγ/LPS i.t at 0h. Some mice were treated with I-BET-762 or vehicle (hydroxyl-β cyclodextrin) i.v 1h before and 4h after IFNγ/LPS exposure. Endpoints were assessed at 12h. Assessment of AHR (A/B) and BALF neutrophil (C) and macrophage counts (D). Representative of three independent experiments, n = 6–8 mice per group, presented as mean ± SEM. *Designates significant differences to PBS-treated controls (**P<0.01, ****P<0.0001), ^#^Designates significant differences from IFNγ/LPS/I-BET-762 treated mice (^##^P<0.01, ^####^P<0.0001).

To determine if BET proteins regulate steroid-resistant airways disease, we first employed our well-established, short-term murine model of IFNγ/LPS-induced steroid-resistant AHR and airway inflammation [12]. As previously reported, AHR was increased following IFNγ/LPS treatment, compared to PBS controls (Fig 1.A and 1.B). Vehicle administration alone did not induce AHR and vehicle administration (control for I-BET-762) in IFNγ/LPS-stimulated mice had no effect on AHR (Fig 1.A and 1.B). Interestingly, I-BET treatment completely abolished IFNγ/LPS-induced AHR (Fig 1.A and 1.B). We have previously shown that treatment of mice with IFNγ or LPS alone do not induce steroid-resistant AHR and inflammation [14].

To assess airway inflammation, leukocyte numbers were quantified in BALF. Neutrophil numbers were dramatically increased after IFNγ/LPS stimulation alone, while macrophage numbers were unchanged (Fig 1.C and 1.D). Vehicle administration in IFNγ/LPS-stimulated mice significantly increased neutrophil numbers, but had no effect on macrophage numbers, compared to those treated with IFNγ/LPS alone (Fig 1.C and 1.D). I-BET-762 treatment effectively reduced neutrophil and macrophage numbers, compared to the IFNγ/LPS/vehicle group (Fig 1.C and 1.D). However, the numbers of macrophages were not significantly different between PBS and IFNγ/LPS/I-BET groups (Fig 1.D). We have previously demonstrated that AHR is dependent on activation of pulmonary macrophages and not neutrophils in this model [14].

**Fig 2:**
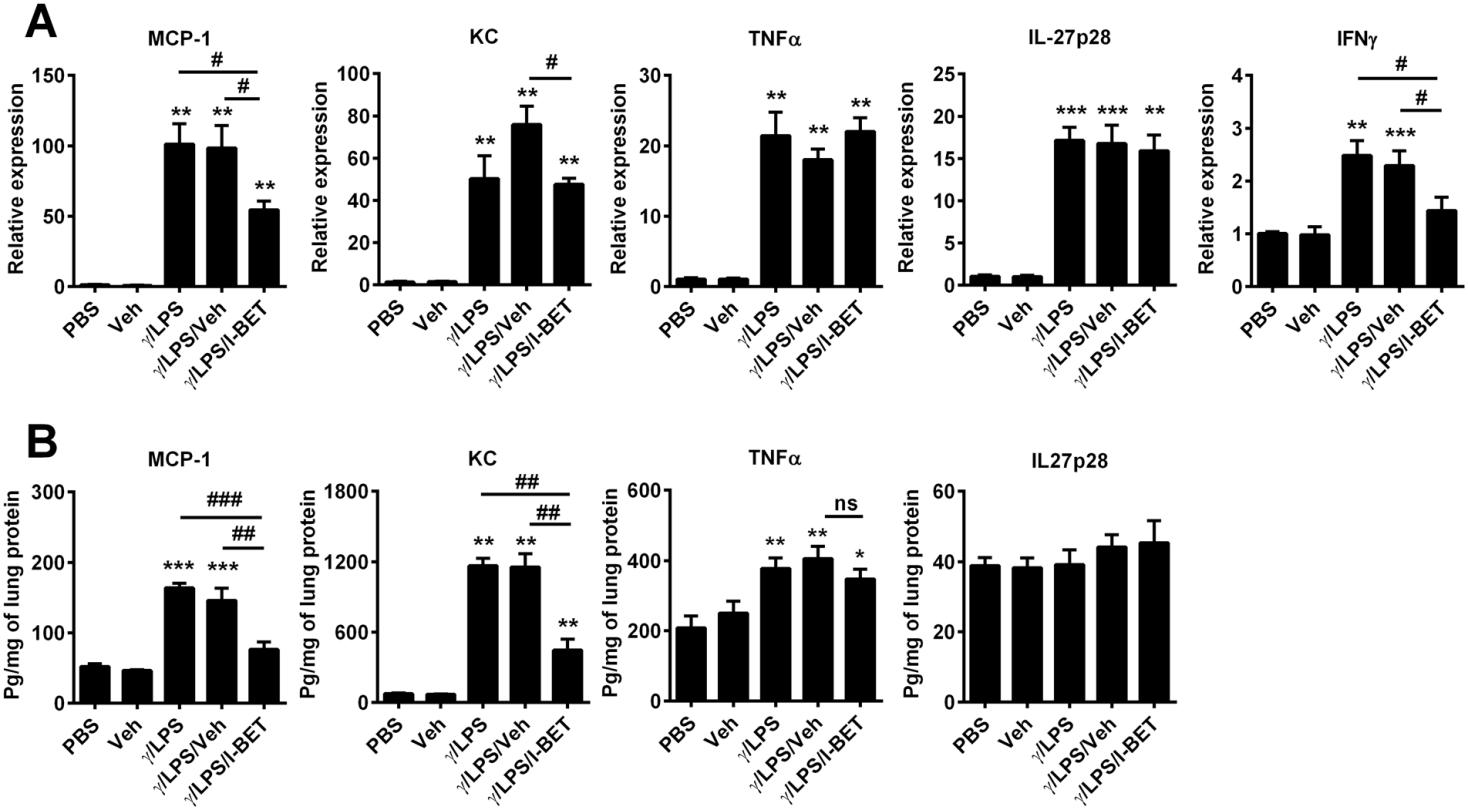
I-BET-762 treatment suppresses lung inflammatory cytokines after IFNγ/LPS stimulation. Effect of I-BET-762 administration on MCP-1, KC, TNFα, IL-27p28 and IFNγ (A) mRNA levels and (B) protein levels in whole lung tissue. Representative of three independent experiments, n = 6–8 mice per group, presented as mean ± SEM. *Designates significant differences to PBS-treated controls (*P<0.05, **P<0.01, ***P<0.001), ^#^Designates significant differences from IFNγ/LPS/I-BET-762 treated mice (^#^P<0.05, ^##^P<0.01, ^###^P<0.001).

Next, we examined the effect of I-BET-762 administration on inflammatory cytokine levels in the lungs. IFNγ/LPS administration significantly increased expression of MCP-1, KC, TNFα, IL-27p28 and IFNγ mRNA in total lung tissue (factors all implicated in the development of steroid resistant inflammation and AHR) (Fig 2.A). I-BET-762 treatment suppressed expression and protein levels of both MCP-1 and KC compared with the IFNγ/LPS/vehicle groups (Fig 2.A and 2.B). IFNγ mRNA levels were also downregulated by I-BET-762 treatment (Fig 2.A), however, we could not meaningfully assess IFNγ protein levels, as recombinant IFNγ is administered to induce this disease model. By contrast, I-BET-762 had no effect on expression of IL-27p28 or TNFα in the lung after IFNγ/LPS stimulation (Fig 2.A and 2.B). Our results indicate that I-BET-762 effectively suppresses IFNγ/LPS-induced steroid-resistant AHR and airway inflammation and inhibits the production of MCP-1 and KC, and the expression of IFNγ in the lung.

**Fig 3:**
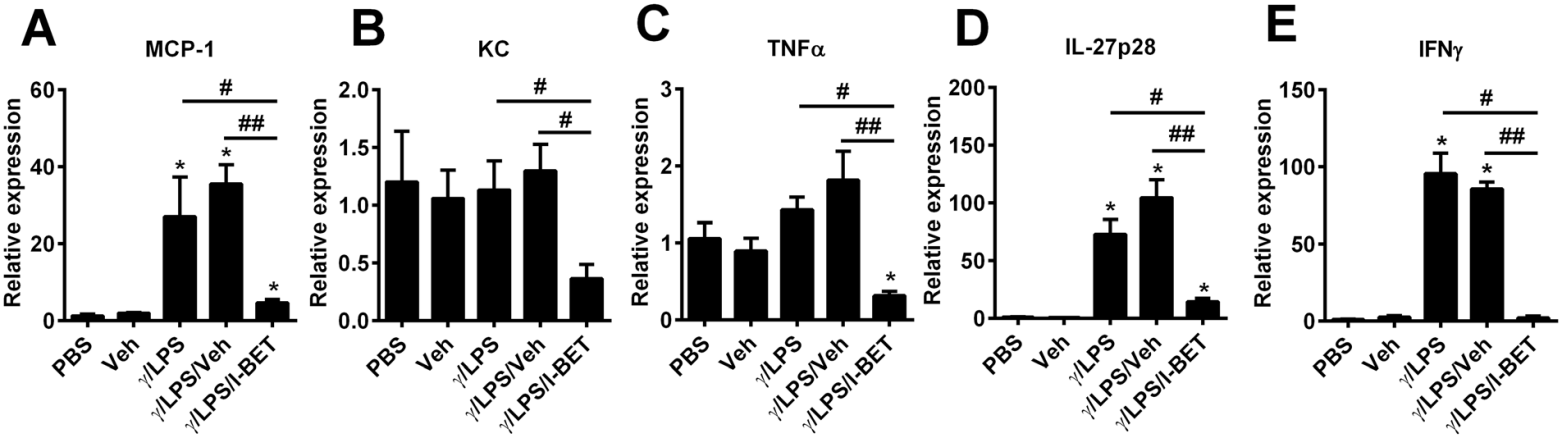
I-BET-762 treatment suppresses inflammatory cytokines/chemokines in IFNγ/LPS-stimulated pulmonary macrophages *in vitro*. Pulmonary macrophages were isolated from naïve mice and stimulated with IFNγ/LPS in presence of I-BET-762 or vehicle for 12h. Expression of MCP-1 (A), KC (B), TNFα (C), IL27p28 (D), and IFNγ (E) were assessed by qPCR. Values are presented as mean ± SEM. *Designates significant differences to PBS-treated controls (*P<0.05), ^#^Designates significant differences to IFNγ/LPS/I-BET-762 treated mice (^#^P<0.05, ^##^P<0.01).

We have previously shown a critical role for macrophages in the development of steroid resistance in our IFNγ/LPS disease model [14]. Thus, we also assessed the effect of I-BET-762 on the production of pro-inflammatory cytokines in IFNγ/LPS-stimulated pulmonary macrophages *in vitro*. Macrophages were isolated from naïve mouse lungs and stimulated with PBS, vehicle alone, or IFNγ/LPS after pre-treatment with either I-BET-762 or vehicle control. Levels of MCP-1, IL-27p28 and IFNγ were markedly increased following IFNγ/LPS stimulation, while KC and TNFα were unchanged from baseline (Fig 3). Pre-treatment with I-BET-762 decreased levels of MCP-1, IL-27, IFNγ, KC and TNFα mRNA compared with IFNγ/LPS/Veh stimulation (Fig 3). Vehicle administration had no effect on gene expression. Our data indicates that I-BET-762 effectively reduced expression of the pro-inflammatory molecules MCP-1, IL-27, IFNγ, KC and TNFα by isolated macrophages after LPS/ IFNγ stimulation.

### MCP-1 plays a critical role in the mechanism of I-BET-762 suppression of steroid resistant inflammation and AHR induced by IFNγ/LPS

**Fig 4:**
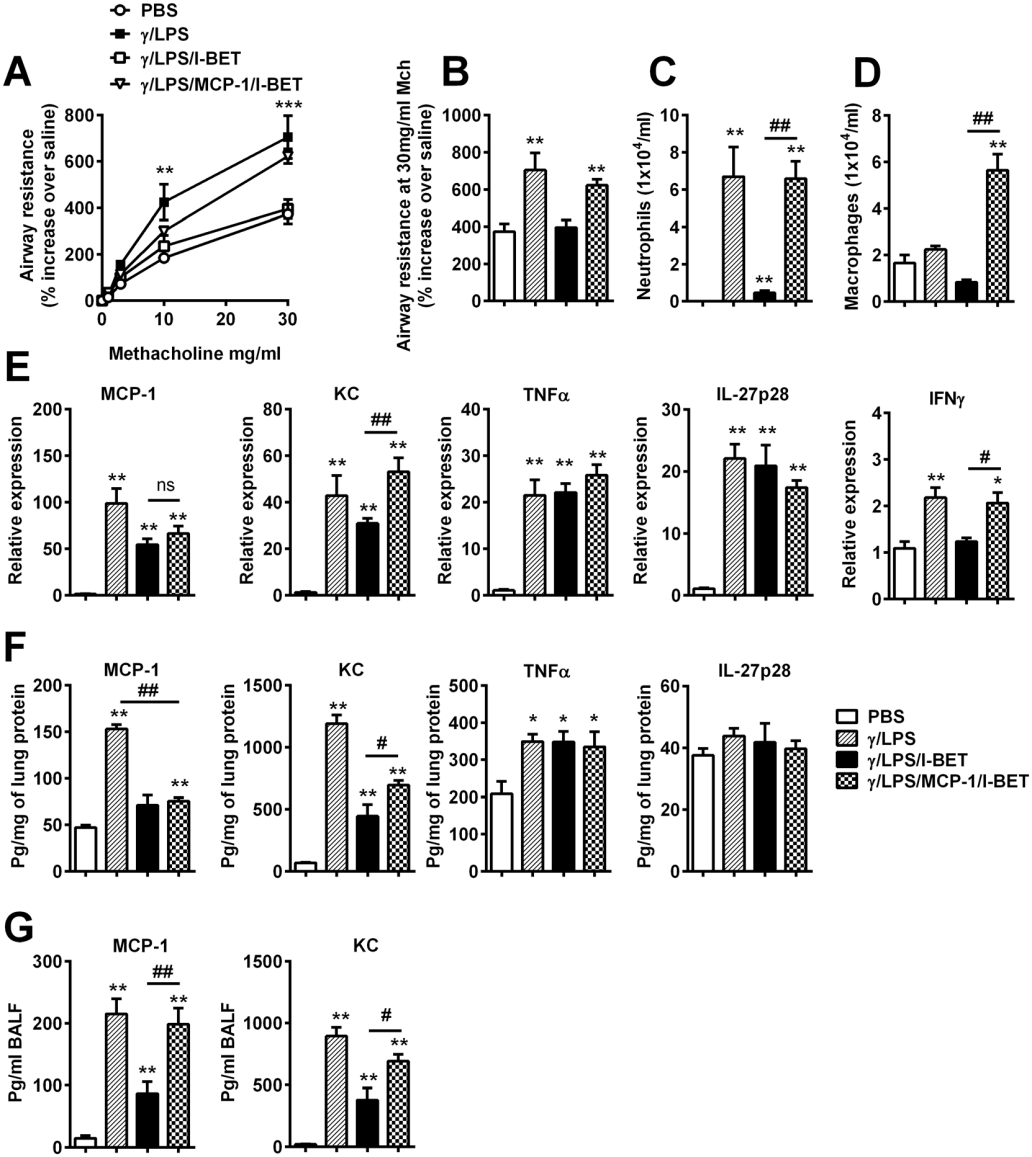
Recombinant MCP-1 administration overcomes I-BET-762-mediated suppression of AHR and inflammation. Mice were administered IFNγ/LPS/rMCP-1 i.t at 0h. Some groups were treated with I-BET-762 i.v 1h before and 4h after IFNγ/LPS/rMCP-1 exposure. Endpoints were assessed at 12h. Effect of I-BET-762 administration on AHR (A/B) and BALF neutrophil (C) and macrophage numbers (D), MCP-1, KC, TNFα, IL-27p28 and IFNγ mRNA levels (A), and protein levels (B) in whole lung tissue, MCP-1 and KC levels in the BALF (D). n = 6–8 mice per group, presented as mean ± SEM. *Designates significant differences to PBS-treated controls (*P<0.05, **P<0.01, ***P<0.001), ^#^Designates significant differences from IFNγ/LPS/MCP-1/I-BET-762-treated mice (^#^P<0.05, ^##^P<0.01).

Macrophages play a key functional role in the development of steroid-resistant pathways induced by IFNγ/LPS [14]. I-BET-762 treatment reduced MCP-1 production in the lung and in isolated macrophages, and also reduced total pulmonary macrophage numbers (Figs 1, 2 and 3). We next assessed whether recombinant MCP-1 administration was sufficient to overcome I-BET-762-mediated protection. In I-BET-762-treated mice, rMCP-1 administration restored AHR to the levels observed after IFNγ/LPS treatment alone (Fig 4.A and 4.B). Neutrophil numbers in the BALF of the IFNγ/LPS/MCP-1/I-BET group were also restored to the levels observed after IFNγ/LPS treatment alone (Fig 4.C). rMCP-1 administration also significantly increased macrophage numbers (to levels greater than IFNγ/LPS treatment alone) in BALF (Fig 4.D). As expected, we observed increased levels of MCP-1 protein in the BALF following rMCP-1 stimulation (Fig 4.G). Expression of KC and IFNγ, but not TNFα and IL-27p28, in the lung tissue were also restored following rMCP-1 administration (Fig 4.E–4.G). These results indicate that MCP-1 administration is sufficient to overcome I-BET-762-mediated suppression of IFNγ/LPS-induced inflammation and AHR.

### I-BET-762 administration inhibits macrophage infiltration and AHR in a mouse model of RSV-induced exacerbations of allergic airways disease

Based on the observation that I-BET-762 suppressed disease in our short-term IFNγ/LPS-driven model, we next assessed the effect of I-BET-762 in a more complex model of viral-induced exacerbation. In previous work, we demonstrated that RSV infection in the context of underlying allergic airways disease exacerbates airway inflammation and AHR [15]. RSV-induced exacerbations were steroid-resistant and associated with increased infiltration of macrophages and neutrophils and elevated levels of cytokines including MCP-1, KC and TNFα in the lung [15].

**Fig 5:**
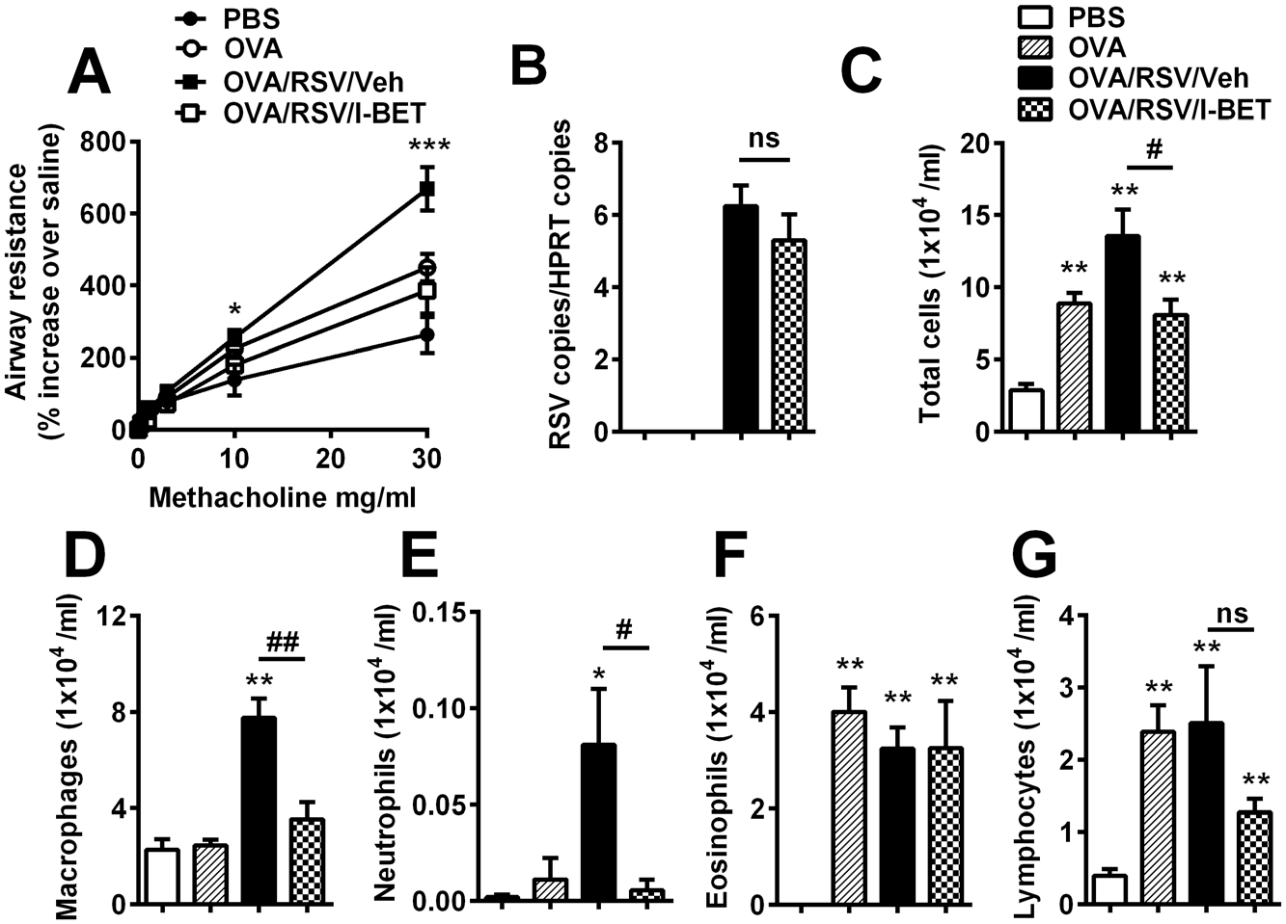
I-BET-762 treatment suppresses AHR and inflammatory cell infiltration in RSV-induced exacerbation. BALB/c mice were sensitised i.p with OVA or PBS plus alum on day 0 and exposed to OVA aerosol on days 13–16. Some mice were inoculated with RSV on day 19 and treated with I-BET-762 or vehicle (hydroxyl-β cyclodextrin) i.v twice on day 23. Lung function and airway inflammation were assessed on day 24. (A) AHR lung assessments, (B) viral copies in total lung tissue and (C) total BALF cell counts. Differential microscopy counts for macrophages (D), neutrophils (E) eosinophils (F), and lymphocyte (G) performed by light microscopy. n = 6–8 mice per group, presented as mean ± SEM. *Designates significant differences to PBS-treated controls (*P<0.05, **P<0.01, ***P<0.001), ^#^Designates significant differences from OVA/RSV/I-BET-762-treated mice (^#^P<0.05, ^##^P<0.01).

Mice were initially sensitised and challenged with OVA to induce allergic airways disease [15]. Some mice were subsequently infected with RSV to induce exacerbations and treated twice with either I-BET-762 or vehicle (20% beta-cyclodextrin, 2% DMSO in 0.9% saline) on day 23 (see methods). Lung function and airway inflammation were assessed on day 24. OVA/RSV/Vehicle-treated mice exhibited heightened AHR compared to the OVA control group (Fig 5.A). I-BET-762 administration significantly suppressed AHR, compared to the OVA/RSV/Vehicle group (Fig 5.A), but had no impact on RSV levels in lung tissue (Fig 5.B). The OVA/RSV/Vehicle group had increased total inflammatory cells in BALF, with increased macrophage and neutrophil numbers, but no change in eosinophil or lymphocyte numbers, compared to the OVA group (Fig 5.C–5.G). Interestingly, I-BET-762 treatment reduced both macrophage and neutrophil numbers, to the levels observed in the OVA group (Fig 5.D and 5.E). I-BET-762 treatment had no significant effect on eosinophil or lymphocyte (albeit reduced) numbers.

**Fig 6:**
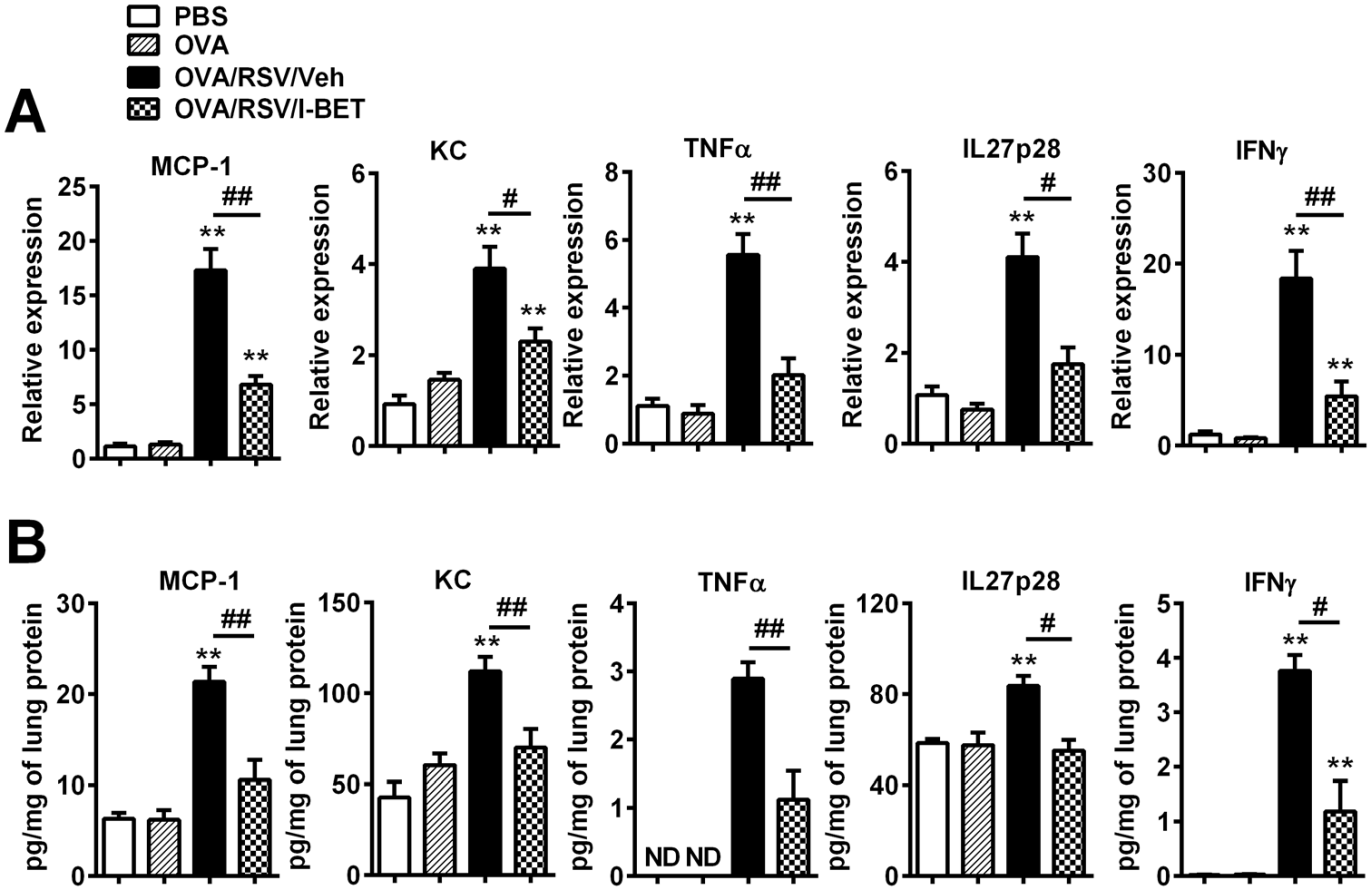
I-BET-762 administration suppresses inflammatory lung cytokines in RSV-induced exacerbation. mRNA levels were assessed by qPCR (A), and protein levels were validated by ELISA (B) on day 24. n = 6–8 mice/group, data presented as mean ± SEM. *Designates significant differences to PBS-treated controls (**P<0.01), ^#^Designates significant differences from OVA/RSV/I-BET-762-treated mice (^#^P<0.05, ^##^P<0.01).

Assessment of lung cytokine levels confirmed that mRNA and protein levels of MCP-1, KC, TNFα, IL27p28 and IFNγ were all significantly increased following RSV-induced exacerbation (Fig 6.A and 6.B). I-BET-762 treatment dramatically decreased mRNA and protein levels of all these cytokines, compared to vehicle treatment (Fig 6.A and 6.B). Expression of the type 1 interferons IFNα and IFNβ were not affected following I-BET-762 treatment (S1.A and S1.B Fig). Further, I-BET-762 treatment had no effect on expression of the eosinophil chemokines eotaxin 1 and eotaxin 2, or the numbers of eosinophils and mucus secreting cells in lung tissue (S1.C–S1.F Fig). Collectively, our results indicate that I-BET-762 effectively suppresses RSV-induced exacerbations by suppressing the levels of inflammatory cytokines that underpin the development of steroid resistant airways inflammation and AHR.

## Discussion

Respiratory viral infections are a major trigger of acute exacerbations of asthma, which result in morbidity and mortality in asthma [3, 5]. Corticosteroid treatment often fails to control the symptoms of asthma exacerbations and effective therapies are urgently needed. I-BET-762 selectively inhibits expression of pro-inflammatory genes by binding to BET proteins and interfering with binding to histone acetylation sites [25, 28, 32]. The ability of the BET inhibitor I-BET-762 to suppress inflammatory gene expression has now been demonstrated in a variety of inflammatory diseases (e.g. cancer and autism-like syndrome) [33–35]. However, it not known whether BET inhibitors have the capacity to suppress the mechanisms that underpin steroid-resistant inflammatory processes that lead to exacerbations of asthma. In this study, we performed proof-of-principle studies and examined the effect of I-BET-762 treatment in two mouse models of steroid-resistant airway inflammation.

Increasing levels of IFNγ and LPS (endotoxin) are directly associated with severity of asthma, and investigations with these factors demonstrate that they drive critical pathogenic mechanisms linked to exacerbations [16, 17, 36]. We have previously demonstrated that cooperative signalling between IFNγ and LPS induces a TLR4/MyD88 dependent steroid-resistant inflammation and AHR, which is dependent on activation of pulmonary macrophages by downstream IL-27 [12]. In this model, I-BET-762 treatment reduced the production of proinflammatory molecules and levels of macrophages that resulted in attenuation of steroid-resistant AHR. Similarly, I-BET-762 treatment attenuated proinflammatory mediator production and the recruitment of neutrophils and macrophages to the lung, which was directly associated with inhibition of steroid-resistant AHR in our model of RSV-induced exacerbation of allergic airways disease. AHR is a key feature of asthma, and the degree of AHR is associated with both disease severity and acute exacerbations [1, 37]. Treatments targeted at reducing inflammation and AHR have demonstrated efficacy for long-term asthma control in the clinic [38]. Importantly, suppression of AHR in our models following I-BET-762 treatment correlates with reduced pulmonary macrophage and neutrophil infiltration as well as expression of the key chemoattractants and/or activators of these cells (e.g. KC, MCP-1, IFNγ and IL-27p28).

While eosinophils are generally associated with allergic asthma, increased neutrophil numbers and high levels of the neutrophil chemokine IL-8 are commonly observed during acute exacerbations of asthma that often poorly respond to standard corticosteroid therapy [5, 6, 39]. In addition, I-BET-762 suppresses IL-8 production by airway smooth muscle cells isolated from asthmatic patients [32]. In this regard, I-BET-762 likely suppresses the production of KC and neutrophil infiltration by blocking the recruitment of BRD proteins. I-BET-762 has been previously demonstrated to inhibit T cell differentiation, but has no effect on mature T cell function [27]. In our IFNγ/LPS and RSV-induced exacerbation models, I-BET treatment is only administered towards the end of the models (12 and 24 hours, respectively) and the timing may be insufficient to impact on T cell differentiation. However, this could be a potential consideration when administering BET inhibitors over longer timeframes.

Activated macrophages also play important roles in steroid-resistant airway inflammation and in acute exacerbations [10, 12, 13, 15]. Macrophage activation is associated with impaired lung function [19], and impaired macrophage function has been observed in subjects with severe asthma [10, 17]. Notably, in our study, both models exhibit macrophage-mediated steroid-resistant AHR and neutrophilic infiltration [12, 15]. The suppression of IFNγ/LPS-induced AHR by I-BET-762 treatment may result from decreased MCP-1 expression, through decreased pulmonary macrophage recruitment and activation. This is supported by our findings that AHR and BALF macrophage infiltration are restored in IFNγ/LPS/I-BET-762 treated mice after administration of MCP-1. Furthermore, we have previously shown that macrophage depletion or neutralisation of MCP-1 significantly inhibits RSV-induced exacerbation of AHR [15]. Therefore, our data suggests that I-BET-762 likely suppresses AHR in both models by targeting pulmonary macrophages and neutrophils, two key inflammatory cells that contribute to the pathogenesis of steroid-resistant AHR. However, we have not assessed the direct effect of MCP-1 on steroid-resistant AHR and airway inflammation. Further, we note that I-BET-762 treatment could also be mediating its effect through other cell types.

In previous studies, we have demonstrated that pulmonary macrophage activation by integrated signalling networks (e.g. IFNγ, TLR4, MyD88 and IL-27) leads to the induction of steroid-resistant AHR in mouse models [12, 14]. In particular, IL-27 (whose production is promoted by IFNγ/LPS administration) underpins disease pathogenesis [12]. Unexpectedly, we did not observe any change of IL-27 production in whole lung homogenates in response to IFNγ/LPS after I-BET-762 treatment. In the lungs of IFNγ/LPS treated mice, macrophages are predominant cellular source of IL-27. As pulmonary macrophages only account a small part of whole lung tissue and the stimulation with IFNγ/LPS is comparably mild and short, it would be difficult to detect significant change of IL-27 in the whole lung tissue. However, we did observe a suppression of IL-27 production in IFNγ/LPS-stimulated pulmonary macrophages *in vitro*, after exposure to I-BET-762. Our observation is similar to a previous study that reported pre-treatment with I-BET-762 could suppress the production of IL-27 in LPS-stimulated BMDMs *in vitro* [25]. In our model of RSV-induced exacerbation, I-BET-762 treatment suppressed multiple proinflammatory factors (including MCP-1, KC, TNFα, IL-27 and IFNγ). We note that we were unable to directly assess whether this suppression occurred through inhibition of BET protein binding to individual gene promoters. Further study will demonstrate which effects are directly caused by I-BET-762 treatment. We previously demonstrated that MCP-1 and TNFα are both produced by macrophages in this model and that blocking either MCP-1 or TNFα function could suppress RSV-induced steroid resistant airway inflammation and AHR [15]. Similar to our current findings, pre-treatment with I-BET-762 has previously been shown to reduce serum IFNγ levels and protect mice in a lethal sepsis induced by LPS [25]. Further, treatment with a different BET inhibitor, JQ1, also inhibited TNFα and MCP-1 expression in LPS-stimulated BMDMs [40] and TNFα levels in a mouse model of *Helicobacter pylori* [26]. Of note, I-BET had no effect on RSV loads or IFNα/β expression in the lungs in our exacerbation model, suggesting that anti-viral responses were not affected and that suppression of the steroid-resistant processes was regulated by the anti-inflammatory effects of I-BET-762.

Collectively, our data demonstrate that I-BET-762 treatment can suppress key features of steroid-resistant airway inflammation and AHR in two mouse models. To our knowledge, this is the first study to show suppressive effects of I-BET-762 on immune pathways that predispose to steroid-resistant inflammation and AHR that underpin exacerbations of asthma. BET inhibitors or a better understanding of how they inhibit inflammation may have potential for the development of therapeutic strategies for the treatment steroid resistant exacerbations of asthma and other chronic obstructive airways diseases where these mechanisms may operate (e.g. chronic obstructive pulmonary disease).

## Supporting Information

S1 Fig I-BET-762 administration has no effect on type 1 interferon or eotaxin expression or numbers of eosinophils and mucus secreting cells in RSV-induced exacerbation. IFNα (A), IFNβ (B), eotaxin-1 (C) and eotaxin-2 (D) mRNA levels in lung tissue were assessed by qPCR on day 24 with the following primer sequences IFNα Fwd 5’-cacagcccagagagtgaccagc-3’, Rev 5’-ggccctcttgttcccgaggt-3’; IFNβ Fwd 5’-ccctatggagatgacggaga-3’, Rev 5’-acccagtgctggagaaattg-3’; Eotaxin-1 Fwd 5’-cccaacacactactgaagagct-3’, Rev 5’-tttgcccaacctggtcttg-3’; Eotaxin-2 Fwd 5’-acggcagcatctgtcccaag-3’, Rev 5’-gtgcctctgaacccacagca-3’. Lung tissues were collected, fixed, sectioned and stained using chromotrope for eosinophil quantification (E) or periodic acid-Schiff for mucus secreting cell (MSC) quantification (F). n = 6–8 mice/group, data presented as mean ± SEM. *Designates significant differences to PBS-treated controls (*P<0.05, **P<0.01).
(TIF)
