# Supplementary figures and images for: Bromodomain and Extra Terminal (BET) Inhibitor Suppresses Macrophage-Driven Steroid-Resistant Exacerbations of Airway Hyper-Responsiveness and Inflammation
Source: PLoS One. 2016 Sep 22;11(9):e0163392. doi: 10.1371/journal.pone.0163392 (PMC5033241; doi:10.1371/journal.pone.0163392)

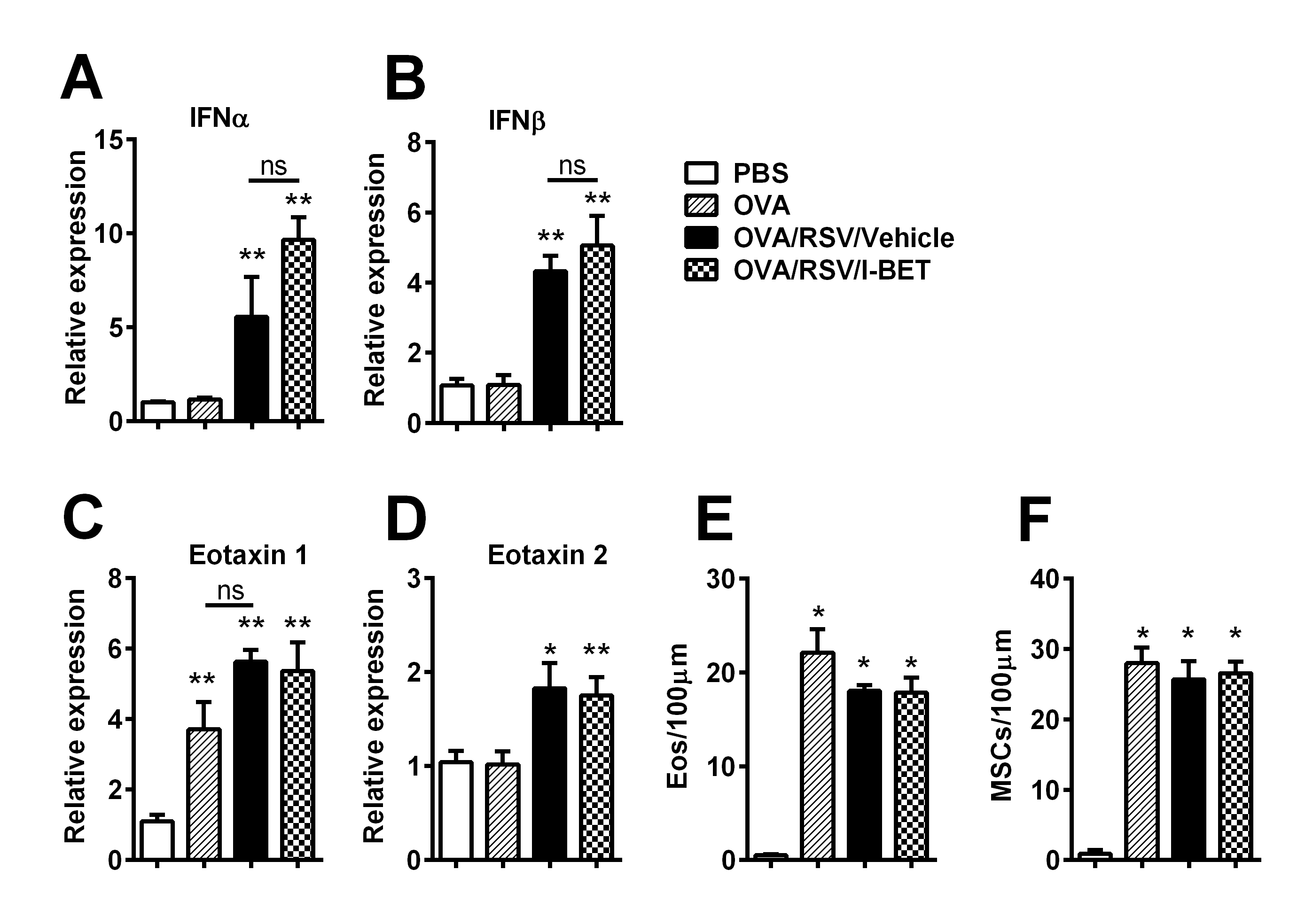

Supplement: S1 Fig — IFNα (A), IFNβ (B), eotaxin-1 (C) and eotaxin-2 (D) mRNA levels in lung tissue were assessed by qPCR on day 24 with the following primer sequences IFNα Fwd 5’-cacagcccagagagtgaccagc-3’, Rev 5’-ggccctcttgttcccgaggt-3’; IFNβ Fwd 5’-ccctatggagatgacggaga-3’, Rev 5’-acccagtgctggagaaattg-3’; Eotaxin-1 Fwd 5’-cccaacacactactgaagagct-3’, Rev 5’-tttgcccaacctggtcttg-3’; Eotaxin-2 Fwd 5’-acggcagcatctgtcccaag-3’, Rev 5’-gtgcctctgaacccacagca-3’. Lung tissues were collected, fixed, sectioned and stained using chromotrope for eosinophil quantification (E) or periodic acid-Schiff for mucus secreting cell (MSC) quantification (F). n = 6–8 mice/group, data presented as mean ± SEM. *Designates significant differences to PBS-treated controls (*P<0.05, **P<0.01). (TIF) [file pone.0163392.s001.tif]
